# Supplementary material for: Evaluation of Subcortical Structure Volumes in Patients with Non-Specific Digestive Diseases
Source: Diagnostics (Basel). 2022 Sep 9;12(9):2199. doi: 10.3390/diagnostics12092199 (PMC9497680; doi:10.3390/diagnostics12092199)
Supplement: Supplementary file 1 [file diagnostics-12-02199-s001.zip › diagnostics-1850879-supplementary.pdf]

*Group

1 - Control

2 - FD

3 - IBD

4 - IBS

*N - group size

*Mean - average

* SD - standard deviation

Group:		N	Mean	SD
1	Left-Cerebellum-White-Matter	19	.009915740283662	.001127732308385
2	Left-Cerebellum-White-Matter	18	.009773215292850	.001097150000982
3	Left-Cerebellum-White-Matter	19	.009586345050427	.000905875982789
4	Left-Cerebellum-White-Matter	20	.009708221479330	.001065787811685

1	Left-Cerebellum-Cortex	19	.036282672730768	.003023968143690
2	Left-Cerebellum-Cortex	18	.036301957179274	.003658612959625
3	Left-Cerebellum-Cortex	19	.034832265745828	.002194033015632
4	Left-Cerebellum-Cortex	20	.035530252173767	.002560890140991

1	Left-Thalamus-Proper	19	.005107293017896	.000373403012209
2	Left-Thalamus-Proper	18	.004986429945082	.000439583347195
3	Left-Thalamus-Proper	19	.004726772088516	.000291279052589
4	Left-Thalamus-Proper	20	.004996306996211	.000240476016762

1	Left-Caudate	19	.002387851514321	.000251576207710
2	Left-Caudate	18	.002366569317607	.000300385800169
3	Left-Caudate	19	.002286836838397	.000159617711690
4	Left-Caudate	20	.002277648613573	.000210541651426

1	Left-Putamen	19	.003364936548116	.000325735790010
2	Left-Putamen	18	.003388893706138	.000327798256289
3	Left-Putamen	19	.003306074728357	.000328950518046
4	Left-Putamen	20	.003239947623685	.000283523698793

1	Left-Pallidum	19	.001398334561045	.000120351051556
2	Left-Pallidum	18	.001343366598431	.000118849663783
3	Left-Pallidum	19	.001300750750645	.000118813164611
4	Left-Pallidum	20	.001347207988809	.000096050995252
1	Left-Hippocampus	19	.002697480723098	.000212428227826
2	Left-Hippocampus	18	.002778845209639	.000329522304822
3	Left-Hippocampus	19	.002707332729804	.000203321617149
4	Left-Hippocampus	20	.002720397115052	.000198728409477
1	Left-Amygdala	19	.001182943797273	.000125012099452
2	Left-Amygdala	18	.001081916076015	.000112959136033
3	Left-Amygdala	19	.001111472944064	.000099057457088
4	Left-Amygdala	20	.001125895850506	.000151863365901
1	Left-Accumbens-area	19	.000314759397257	.000048422934235
2	Left-Accumbens-area	18	.000320559030077	.000067245539021
3	Left-Accumbens-area	19	.000322505393762	.000056512799776
4	Left-Accumbens-area	20	.000340180889333	.000064437229466
1	CSF	19	.000605570587393	.000092409950956
2	CSF	18	.000637292940662	.000134332048019
3	CSF	19	.000592762762630	.000113600602481
4	CSF	20	.000602510291082	.000122005141297
1	Right-Cerebellum-White-Matter	19	.009081480265598	.001056668002603
2	Right-Cerebellum-White-Matter	18	.009122593921823	.000962745719428
3	Right-Cerebellum-White-Matter	19	.008966959962808	.000687647972269
4	Right-Cerebellum-White-Matter	20	.009224154456250	.000999608615553
1	Right-Cerebellum-Cortex	19	.036520892154109	.003213588823342
2	Right-Cerebellum-Cortex	18	.036436486712713	.003690672548429
3	Right-Cerebellum-Cortex	19	.035625452203870	.002261853126784
4	Right-Cerebellum-Cortex	20	.036157001533814	.002636072579246
1	Right-Thalamus-Proper	19	.004883977665169	.000300641686875
2	Right-Thalamus-Proper	18	.004827191603355	.000346929334407

3	Right-Thalamus-Proper	19	.004587687140537	.000324333872869
4	Right-Thalamus-Proper	20	.004753271585739	.000239186190948
1	Right-Caudate	19	.002443349858320	.000235699113626
2	Right-Caudate	18	.002482389071934	.000284838499321
3	Right-Caudate	19	.002327649744600	.000132863013715
4	Right-Caudate	20	.002357721832159	.000237968276775
1	Right-Putamen	19	.003360311279612	.000299270491304
2	Right-Putamen	18	.003458550010718	.000347682171897
3	Right-Putamen	19	.003346270193051	.000286536518937
4	Right-Putamen	20	.003301588761964	.000279710546259
1	Right-Pallidum	19	.001359548793953	.000117403980664
2	Right-Pallidum	18	.001328160988541	.000120432680575
3	Right-Pallidum	19	.001279996853697	.000109064134290
4	Right-Pallidum	20	.001330834961916	.000101139589091
1	Right-Hippocampus	19	.002824836424406	.000219495334670
2	Right-Hippocampus	18	.002841012470798	.000233919731519
3	Right-Hippocampus	19	.002817714526869	.000211587837320
4	Right-Hippocampus	20	.002846532673904	.000212176268778
1	Right-Amygdala	19	.001246231336089	.000119339174714
2	Right-Amygdala	18	.001190046299284	.000100151019236
3	Right-Amygdala	19	.001183378600650	.000079853101880
4	Right-Amygdala	20	.001195907477623	.000119525742568
1	Right-Accumbens-area	19	.000336286337650	.000043119086879
2	Right-Accumbens-area	18	.000360172230867	.000061294707780
3	Right-Accumbens-area	19	.000348198927775	.000052366668501
4	Right-Accumbens-area	20	.000358497188629	.000049290339953
1	WM-hypointensities	19	.000473917065940	.000145358660888
2	WM-hypointensities	18	.000516928405608	.000120847739337
3	WM-hypointensities	19	.000490749539646	.000210379998900
4	WM-hypointensities	20	.000477403939566	.000129447303367

1	CC_Posterior	19	.000645391864617	.000127954328787
2	CC_Posterior	18	.000618054084680	.000049801932934
3	CC_Posterior	19	.000588482615703	.000091709295743
4	CC_Posterior	20	.000649042602655	.000100499819342
1	CC_Mid_Posterior	19	.000376594263370	.000072161525973
2	CC_Mid_Posterior	18	.000373041078811	.000057821876168
3	CC_Mid_Posterior	19	.000345588882955	.000048197688724
4	CC_Mid_Posterior	20	.000363168687117	.000069413371387
1	CC_Central	19	.000386321372404	.000090397672937
2	CC_Central	18	.000389692711180	.000115102763543
3	CC_Central	19	.000368231285605	.000067371404632
4	CC_Central	20	.000431782791532	.000084890251427
1	CC_Mid_Anterior	19	.000412349578366	.000087309923862
2	CC_Mid_Anterior	18	.000365608892347	.000072430296087
3	CC_Mid_Anterior	19	.000396629615127	.000096315771789
4	CC_Mid_Anterior	20	.000441356505695	.000130205109472
1	CC_Anterior	19	.000618440754319	.000103643245946
2	CC_Anterior	18	.000599807424621	.000058398228267
3	CC_Anterior	19	.000582202253124	.000106865378879
4	CC_Anterior	20	.000612906435229	.000078850512317
1	BrainSegVol	19	.753403534883892	.017838828963752
2	BrainSegVol	18	.749508229494214	.018376830145382
3	BrainSegVol	19	.745197959429032	.022744768719438
4	BrainSegVol	20	.749131380748355	.016474455868007
1	BrainSegVolNotVent	19	.741372809333531	.018798104619581
2	BrainSegVolNotVent	18	.736555634602148	.020766723734005
3	BrainSegVolNotVent	19	.733289440220927	.024001666080552
4	BrainSegVolNotVent	20	.738573034595273	.018364392229368

1	lhCortexVol	19	.153392517407796	.006203228048493
2	lhCortexVol	18	.154438793057625	.006659706952337
3	lhCortexVol	19	.151118042413795	.006355745798369
4	lhCortexVol	20	.153128638729331	.008706044785447
1	rhCortexVol	19	.154897529540881	.006902131731211
2	rhCortexVol	18	.155151799006505	.006716537999786
3	rhCortexVol	19	.151608058212633	.006944658101006
4	rhCortexVol	20	.154667973881245	.008590111811204
1	CortexVol	19	.308290046948585	.012980424753189
2	CortexVol	18	.309590592064091	.013220225369503
3	CortexVol	19	.302726100626434	.013175714937606
4	CortexVol	20	.307796612610849	.017151963042698
1	lhCerebralWhiteMatterVol	19	.151012073931378	.007481784079595
2	lhCerebralWhiteMatterVol	18	.148414379874533	.007370942255889
3	lhCerebralWhiteMatterVol	19	.151803243341890	.010294559912461
4	lhCerebralWhiteMatterVol	20	.150578837690202	.007418222251893
1	rhCerebralWhiteMatterVol	19	.151033763010266	.007568947419739
2	rhCerebralWhiteMatterVol	18	.147927177926386	.007379909839317
3	rhCerebralWhiteMatterVol	19	.151808149938394	.009754458014663
4	rhCerebralWhiteMatterVol	20	.150747198348568	.007269644431587
1	CerebralWhiteMatterVol	19	.302045836941504	.014999967478932
2	CerebralWhiteMatterVol	18	.296341557801065	.014662759627106
3	CerebralWhiteMatterVol	19	.303611393280388	.019978350106545
4	CerebralWhiteMatterVol	20	.301326036038711	.014613480981549
1	SubCortGrayVol	19	.039301294206342	.001941030180333
2	SubCortGrayVol	18	.039017779388110	.002694959019962
3	SubCortGrayVol	19	.037798603780658	.001897880240920
4	SubCortGrayVol	20	.038549574510653	.001617150078715
1	TotalGrayVol	19	.421049189547565	.013758381641986
2	TotalGrayVol	18	.421889141300774	.017099709975430

3	TotalGrayVol	19	.411683782039615	.013133438708485
4	TotalGrayVol	20	.418836051116248	.018675897013687
